# Bigger challenges, similar outcomes: Robotic prostatectomy in the obese patient

**DOI:** 10.1002/bco2.70112

**Published:** 2025-11-09

**Authors:** Andrew Evans, Ibrahim Ibrahim, Thomas Miller, Assia Djoudi, Katharine Hill, Imran Ahmad

**Affiliations:** ^1^ Department of Urology Queen Elizabeth University Hospital Glasgow UK; ^2^ School of Cancer Sciences University of Glasgow, Garscube Estate Glasgow UK; ^3^ CRUK Scotland UK Institute, Garscube Estate Glasgow UK

**Keywords:** functional outcomes, obesity, oncology, prostate cancer, robotic surgery

## Abstract

**Objectives:**

The study aims to review the safety of performing robotic‐assisted radical prostatectomy (RARP) in patients with a body mass index (BMI) > 35 kg/m^2^ in a high‐volume robotic centre.

**Materials and methods:**

A prospective database of all patients who underwent RARP between December 2015 and October 2024 was reviewed. Propensity score matching was done preoperatively on age, prostate‐specific antigen, ISUP grade and T stage. Matched cohort analysis was conducted comparing outcomes in 89 patients with BMI ≥ 35 kg/m^2^ and those with BMI 18–25 kg/m^2^. Outcomes included operational time, estimated blood loss (EBL), positive surgical margins (PSM), complications, length of stay, continence and erectile function at 12 months.

**Results:**

Console time was significantly longer in the high‐BMI group (146 ± 48 min vs. 129 ± 44 min, *p* = 0.02). EBL was also greater (median 350 ml vs. 200 ml, *p* < 0.001). However, there was no significant difference in hospital stay (median 3 days for both groups, *p* = 0.86), nerve sparing rates or PSM. Patients in the obese cohort experienced more complications although this was not statistically significant. At 12 months post‐operatively, continence was comparable between the groups. Median pad use was 1/day (interquartile range [IQR] 0–2) in the obese cohort versus 0/day (IQR 0–1) in the non‐obese cohort (*p* = 0.09). Pad‐free status was achieved in 48.3% compared with 61.8% respectively (*p* = 0.06). Erectile function recovery found 14.8% regaining function in the obese cohort compared with 18.0% in the non‐obese cohort (*p* = 0.82).

**Conclusion:**

This matched cohort analysis demonstrates that obese patients undergoing RARP experience longer operative times and increased EBL. These factors do not adversely impact functional or oncological outcomes. The incidence of post‐operative complications remained low and comparable with patients with a normal BMI. With appropriate surgical expertise, high BMI alone should not be considered a contraindication to RARP.

## 1 INTRODUCTION

Prostate cancer is the second most commonly diagnosed cancer and the fifth leading cause of cancer‐related death among men globally, with over 1.4 million new cases and approximately 375 000 deaths reported in 2020 alone.^1^, ^2^ Internationally, there is an obesity epidemic with one in eight adults now obese. The UK government office for science projects that 60% of adult men in the United Kingdom will be obese by 2050.^3^, ^4^

Obesity has been implicated in both the incidence and progression of various malignancies, including prostate cancer. Though the association between obesity and prostate cancer risk remains complex and sometimes contradictory, numerous studies suggest that higher body mass index (BMI) is linked to more aggressive disease, delayed diagnosis due to lower prostate‐specific antigen (PSA) sensitivity, and worse oncological outcomes.^5^, ^6^, ^7^, ^8^ In addition, obesity has been associated with increased surgical risk, potentially impacting the safety and efficacy of radical prostatectomy, a standard treatment option for localised prostate cancer.

Robotic‐assisted radical prostatectomy (RARP) has largely replaced open or laparoscopic prostatectomy due to its advantages in visualisation, precision and postoperative recovery. However, performing RARP in patients with a BMI ≥ 35 kg/m^2^ poses unique technical and physiological challenges, potentially increasing operative time, blood loss, difficult port placement and limited workspace within the pelvis. These factors may impact perioperative safety as well as long‐term functional and oncological outcomes, such as urinary continence, sexual function and biochemical recurrence. Nevertheless, emerging data suggest that with appropriate surgical expertise, RARP can be safely and effectively performed in this high‐risk population.^6^, ^7^

Despite a limited evidence base, patients with obesity are often steered away from surgical management and instead directed towards radiotherapy, potentially due to perceived technical complexity and elevated perioperative risk.

Given the global rise in obesity and its intersection with prostate cancer, there is an urgent need to clarify the safety and outcomes of RARP in patients with morbid obesity. The aim of this study is to evaluate the perioperative, functional and oncological outcomes of RARP in patients with a BMI > 35 kg/m^2^ in a high‐output robotic department comparing this with patients with a normal BMI (18–25 kg/m^2^).

## 2 MATERIALS AND METHODS

Data from our prospectively maintained RARP database were used for analysis and identified 1931 patients who underwent a RARP between December 2015 and October 2024. All procedures were performed at a single high‐volume robotic centre, with an annual caseload exceeding 250 procedures. In total, four consultant surgeons served as primary operators, including two senior surgeons, each with experience exceeding 1000 procedures. Interrogation of this database isolated 110 patients with BMI > 35 kg/m^2^. Due to incomplete datasets regarding functional follow‐up, 89 patients were included. Four patients had a BMI > 40 kg/m^2^. A 1:1 matched cohort analysis was conducted comparing postoperative outcomes in patients with BMI ≥ 35 kg/m^2^ and BMI 18–25 kg/m^2^ who underwent robotic prostatectomy. Propensity score matching was completed preoperatively based on key clinical and pathological features including age, PSA, ISUP grade and T stage.

We analysed post‐operative variables including hospital stay, console time, estimated blood loss (EBL), nerve sparing performed and positive surgical margins (PSM). Complications recorded were from those seen within the first 30 days post‐operatively. Patients' daily continence pad usage was recorded with continence defined as the use of 0 pads. Erectile function was recorded at 12 months with patients reporting if achieving a satisfactory erection for intercourse with or without the aid of medical therapy.

All data processing and statistical analysis were performed using Python (v3.11). Visualisations were generated using matplotlib. Continuous variables (e.g., BMI, pad usage, EBL, hospital stay and console time) were assessed for normality using the Shapiro–Wilk test. Parametric variables were analysed using paired *t* tests while non‐parametric variables were compared using the Wilcoxon signed‐rank test. Binary categorical outcomes (e.g., erectile function, nerve sparing and surgical margin status) were compared using McNemar's test. Categorical variables, such as Clavien–Dindo complication grade, were compared using the *χ* ^2^ test. A two‐tailed *p* value <0.05 was considered statistically significant. All statistical analyses were performed with appropriate matching accounted for.

## 3 RESULTS

A total of 89 matched patient pairs were included in the analysis. The two groups were well matched in terms of age and disease characteristics, with the only intended difference being BMI (mean BMI 36.6 ± 1.5 vs BMI 22.7 ± 1.5, *p* < 0.001). See Table 1.

**TABLE 1:** Demographic summary of patients included in the study.

Variable	Normal BMI (18–25)	High BMI (≥35)	Standardised mean difference after 1:1 PS matching
Age	63.0 (59.0–67.0)	65.0 (60.0–68.0)	0.291
PSA	9.1 (5.1–12.6)	9.2 (5.6–12.8)	0.015
T2	37 (41.6%)	43 (48.3%)	0.152
T3	41 (46.1%)	46 (51.7%)	
ISUP Grade 1	9 (10.1%)	3 (3.4%)	0.111
ISUP Grade 2	49 (55.1%)	56 (62.9%)	
ISUP Grade 3	22 (24.7%)	24 (27.0%)	
ISUP Grade 4	6 (6.7%)	3 (3.4%)	
ISUP Grade 5	3 (3.4%)	3 (3.4%)	

Abbreviations: BMI, body mass index; PSA, prostate‐specific antigen.

### 3.1 Operative and perioperative outcomes

**FIGURE 1:**
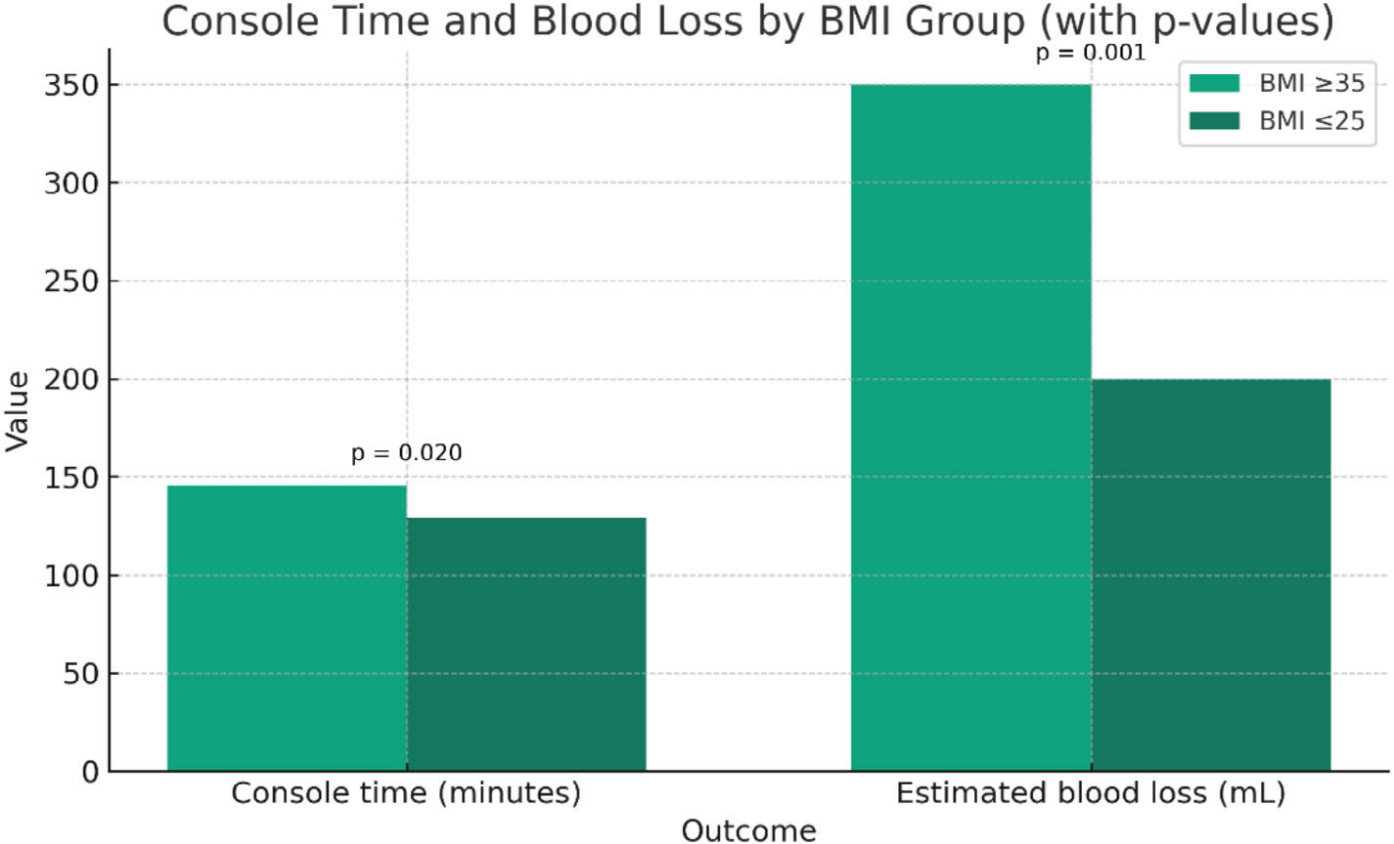
Console time and blood loss by body mass index (BMI) group.

Console time was significantly longer in the obese cohort (146 ± 48 min vs. 129 ± 44 min, *p* = 0.02). EBL was also greater in our obese patient cohort (median 350 ml vs. 200 ml, *p* < 0.001). See Figure 1 and Table 2. There was no significant difference in hospital stay (median 3 days for both groups, *p* = 0.86).

**TABLE 2:** Comparison of key outcomes between BMI groups.

Outcome	BMI ≥ 35 (*n* = 89)	BMI ≤ 25 (*n* = 89)	*p* value (test)
Pads at 12 months (count/day)	1 (0–2)	0 (0–1)	0.09 (Wilcoxon)
Pad‐free at 12 months	48.3% (43/89)	61.8% (55/89)	0.06 (McNemar)
Erectile function at 12 months	14.8% (13/88)	18.0% (16/89)	0.82 (McNemar)
Hospital stay (days)	3 (1–4)	3 (2–3)	0.86 (Wilcoxon)
Console time (min)	146 ± 48	129 ± 44	0.02 (paired t‐test)
Estimated blood loss (mL)	350 (250–500)	200 (150–300)	<0.001 (Wilcoxon)
Nerve sparing performed	42.7% (38/89)	32.6% (29/89)	0.18 (McNemar)
Clavien–Dindo complication grade			
0	80 (89.9%)	86 (96.6%)	0.99 (*χ* ^2^)
1	6 (6.7%)	1 (1.1%)	
2	1 (1.1%)	2 (2.2%)	
3	2 (2.2%)	0 (0%)	
Positive surgical margins	28.1% (25/89)	25.0% (22/88)	0.86 (McNemar)

Abbreviation: BMI, body mass index.

### 3.2 Functional outcomes

Continence at 12 months, as assessed by pad usage, was similar between the groups. Median pad use in the obese cohort was 1 pad per day (IQR 0–2) compared with 0 pads per day (IQR 0–1) in our normal BMI cohort (*p* = 0.09). Additionally pad‐free status at 1 year was achieved in 48.3% of patients with obesity compared with 61.8% in patients without obesity (*p* = 0.06).

**FIGURE 2:**
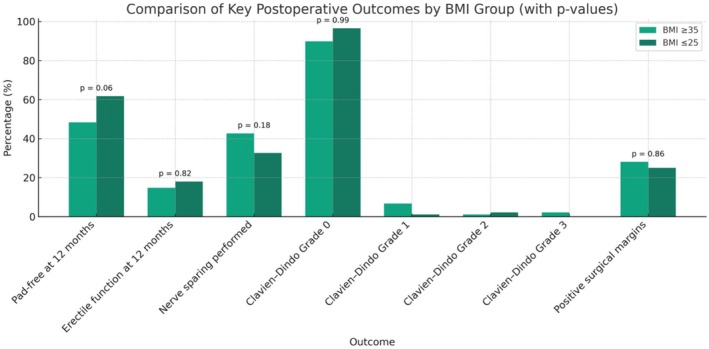
Comparison of key postoperative outcomes by body mass index (BMI) group.

Erectile function, satisfactory for intercourse at 12 months, was also similar: 14.8% (BMI > 35 kg/m^2^) versus 18.0% (BMI < 25 kg/m^2^) (*p* = 0.82). See Figure 2 and Table 2.

### 3.3 Nerve sparing and complications

Nerve sparing was performed in 38/89 (42.7%) of high‐BMI patients and 29/89 (32.6%) of normal BMI patients (*p* = 0.18). See Figure 2 and Table 2.

Postoperative complications, assessed using the Clavien–Dindo classification, were similar between the normal BMI and obese groups. In the obese cohort, 80 patients (89.9%) had no complications (Grade 0), while 6 patients (6.7%) experienced Grade I complications, 1 patient (1.1%) had a Grade II complication and 2 patients (2.2%) had Grade III complications. In the non‐obese cohort, 86 patients (96.6%) had no complications, 1 patient (1.1%) experienced a Grade I complication and 2 patients (2.2%) had Grade II complications; no patients in this group experienced Grade III complications. There was no statistically significant difference in the distribution of complication grades between the groups (*p* = 0.99). See Figure 2 and Table 2.

### 3.4 PSMs

PSM rates were comparable: 28.1% versus 25.0% in our obese and normal‐sized cohorts respectively (*p* = 0.86). See Figure 2 and Table 2.

## 4 DISCUSSION

This matched cohort study investigated the impact of morbid obesity (BMI ≥ 35 kg/m^2^) on perioperative, functional and oncological outcomes following RARP. Despite increased intraoperative complexity, as demonstrated by significantly longer console times and greater blood loss, severe obesity did not adversely affect early oncologic outcomes or key functional parameters at 12 months postoperatively. These findings support the growing body of evidence indicating that high BMI, when managed within the context of experienced surgical teams and high‐volume centres, should not be considered a contraindication to RARP.^6^, ^7^

### 4.1 Perioperative outcomes and surgical complexity

As anticipated, operative time and estimated blood loss were significantly higher in the obese cohort. This aligns with local and international studies and meta‐analyses that report increased intraoperative challenges in obese patients attributed to patient factors including greater abdominal wall thickness, a narrower pelvic cavity and excess periprostatic adiposity. Vascularised adipose tissue in particular can complicate dissection and haemostasis, prolonging operative time.^9^, ^10^ Although blood loss was significantly higher in the obese cohort, the absolute volume rarely approached thresholds requiring clinical intervention such as transfusion, suggesting that this finding is of academic interest with limited practical consequence.

The slightly increased complication rate in obese patients is particularly related to respiratory infections and two cases of returns to theatre, is comparable with what is reported in large robotic series. The physiological impact of prolonged Trendelenburg positioning and pneumoperitoneum in very obese patients contributes to higher respiratory risk and reinforces the significance of anaesthetic optimisation and postoperative recovery protocols in this subgroup.

### 4.2 Functional outcomes: Continence and potency

The recovery of urinary continence and erectile function at 12 months was broadly comparable between the two cohorts analysed. Although the pad‐free rate was lower in the obese cohort (46.3% vs. 61.8%), the difference was not statistically significant. This finding reiterates prior studies that obesity may be associated with delayed, but not absent continence recovery. Prior evidence has reviewed continence recovery at different timescales; however our study captured the pad usage solely at 12 months.

Similarly, erectile function recovery, defined as erections sufficient for satisfactory intercourse, was not significantly different between the cohorts. The overall recovery rate was lower than published series, likely reflecting the use of a stringent, patient‐reported outcome measure rather than questionnaire‐derived validated scoring systems such as the SHIM or IIEF.

### 4.3 Oncological outcomes: PSMs

No significant difference was observed in PSM rates between the cohorts. This is consistent with the literature indicating that BMI is not an independent predictor of margin status nor future biochemical recurrence and progression. Such findings reassure that a high BMI alone does not compromise oncological outcomes.

### 4.4 Comparison with wider literature

Previous studies have reported varied results with respect to intraoperative and functional outcomes.^9^ Our findings contribute to this ongoing debate by extending the evidence base regarding the safety of RARP in patients with higher BMI. Importantly, the appropriate BMI threshold for safe surgery remains uncertain. Although we observed acceptable perioperative and functional outcomes across BMI groups, it should be noted that only a small number of patients in our cohort had a BMI greater than 40, limiting the strength of subgroup analysis.

Nevertheless, this study updates the current understanding of the upper limit of BMI for RARP, demonstrating that with experienced surgeons, anaesthetists and a coordinated multidisciplinary team in a high‐volume robotic centre, RARP can be performed safely in patients with a BMI of 35 or greater. This represents a shift in the perceived boundary of surgical feasibility and highlights the importance of institutional expertise in managing higher‐risk patients.

## 5 LIMITATIONS

This study has several limitations. First, it is a retrospective analysis and therefore subject to inherent biases such as selection and information bias. Although propensity score matching was conducted using key clinical and pathological variables, unmeasured factors—such as comorbidities, patient motivation and baseline functional status—may influence the outcomes. It is also possible that patients with severe obesity who were deemed suitable for RARP represent a relatively fitter subset, introducing a degree of selection bias. Obesity is intrinsically linked to metabolic syndrome and baseline erectile dysfunction, both of which independently affect functional outcomes. This makes it challenging to isolate the direct impact of RARP on postoperative recovery in this population.

Furthermore, functional outcomes were assessed using patient‐reported criteria rather than validated tools, which limits comparability with other published data. Moreover, this study was conducted at a single high‐volume centralised department with significant surgical expertise; thus, the findings may not be generalisable to other centres. Lastly, long‐term oncological endpoints such as biochemical recurrence and survival were not evaluated in these analyses.

## 6 CONCLUSION

In summary, RARP in patients with BMI ≥ 35 kg/m^2^ is feasible and safe, with comparable oncological and functional outcomes to non‐obese patients. Although obese patients face increased intraoperative challenges leading to longer console times and higher blood loss, these factors do not translate into inferior outcomes. RARP can be safely and effectively offered to patients with severe obesity provided appropriate patient counselling and surgical planning.

## AUTHOR CONTRIBUTIONS

The project was completed due to the following contributions. Andrew Evans performed the data collection and wrote the paper. Ibrahim Ibrahim analysed the data and wrote the paper. Thomas Miller contributed to the data analysis and the writing of the paper. Assia Djoudi contributed to data analysis and writing of the paper. Katharine Hill contributed to the data analysis and writing of the paper. Professor Imran Ahmad designed the study's aims, methodology and oversaw all aspects of the study.

## CONFLICT OF INTEREST STATEMENT

No conflicts exist for any authors.
